# Alarming low physical activity levels in Chilean adults with disabilities during COVID-19 pandemic: a representative national survey analysis

**DOI:** 10.3389/fpubh.2023.1090050

**Published:** 2023-06-02

**Authors:** Matías Henríquez, Rodrigo Ramirez-Campillo, Carlos Cristi-Montero, Raul Reina, Cristián Alvarez, Gerson Ferrari, Nicolas Aguilar-Farias, Kabir P. Sadarangani

**Affiliations:** ^1^Escuela de Kinesiología, Facultad de Odontología y Ciencias de la Rehabilitación, Universidad San Sebastián, Providencia, Chile; ^2^Faculty of Rehabilitation Sciences, School of Physical Therapy, Exercise and Rehabilitation Sciences Institute, Universidad Andres Bello, Santiago, Chile; ^3^IRyS Group, Physical Education School, Pontificia Universidad Católica de Valparaíso, Valparaíso, Chile; ^4^Sports Research Centre, Miguel Hernández University, Elche, Spain; ^5^Universidad de Santiago de Chile (USACH), Escuela de Ciencias de la Actividad Física, el Deporte y la Salud, Santiago, Chile; ^6^Department of Physical Education, Sports and Recreation, Universidad de La Frontera, Temuco, Chile; ^7^Escuela de Kinesiología, Universidad Autónoma de Chile, Santiago, Chile; ^8^Faculty of Health and Dentistry, School of Kinesiology, Universidad Diego Portales, Santiago, Chile

**Keywords:** disabled persons, sports for persons with disabilities, COVID-19, Latin America, socioeconomic factors, physical activity

## Abstract

**Background:**

People with disabilities usually face barriers to regularly engaging in physical activities. Estimating physical activity patterns are necessary to elaborate policies and strategies to facilitate active lifestyles, considering the particular access difficulties experienced by this population.

**Purpose:**

This study aimed (i) to describe the prevalence of physical activity levels and (ii) to examine the associations of physical activity levels with socio-demographic variables and type of disability in the 2020 Chilean National Physical Activity and Sports Habits in Populations with Disabilities (CNPASHPwD) survey during the coronavirus disease 19 (COVID-19) pandemic.

**Methods:**

Cross-sectional data from 3,150 adults (18–99 years old), 59.8% female, were analyzed from November to December 2020. Self-reported age, gender, type of disability (i.e., physical, visual, hearing, intellectual, or mixed), socio-economic status, area and zone of residence, and physical activity levels (0 min/week, < 150 min/week, ≥ 150 min/week) were obtained.

**Results:**

11.9% of the participants were classified as active (≥ 150 min/week), and 62.6% declared no involvement in physical activity. A larger proportion of females (61.7%) did not meet the current guidelines (≥ 150 min/week of physical activity) in comparison with males (*p* < 0.001). Participants with visual and hearing disabilities were more likely to be active than those with other types of disabilities. Those living in the central and southern regions of Chile were more likely to be physically active than those from the northern region. Also, older participants, women, and those from lower socio-economic statuses were less likely to meet the physical activity guidelines.

**Conclusion:**

Alarmingly, nine out of ten participants were categorized as physically inactive, particularly women, older adults, and those with a low socioeconomic status. If the pandemic context moderated, the considerable prevalence of reduced physical activity levels deserves future exploration. Health promotion initiatives should consider these aspects, emphasizing inclusive environments and increasing opportunities to favor healthy behaviors, countering the COVID-19 effects.

## 1. Introduction

Inadequate physical activity (PA) levels and sedentary behaviors are linked to non-communicable diseases and all-cause mortality in the global population (1–4). Compared to the average population, people with different disabilities show even lower PA levels and increased risk of comorbidities (5). The benefits of being physically active reinforce the need for providing opportunity access to perform PA to everyone of all ages (6–8). Although people with disabilities face several barriers that limit their participation in PA and which differ from those without disabilities, challenging the possibilities to ensure participation and the benefits of regular practice in this group is imperative (9–11). Global efforts to promote PA in people living with disabilities resulted in the first World Health Organization (WHO) guidelines based on wide-ranging evidence and describing recommendations that could facilitate the practice and participation of overall society activities in this specific population (12).

Most population data to estimate or monitor PA patterns are conducted through national surveys, where the available data are mainly focused on high-income countries or northern regions (North America and Europe) (13). Thereby, a lack of evidence is appreciable in low-to-middle-income countries or Latin-American and African regions. For example, in Netherlands and Australia, adults living with disabilities had lower PA levels and were less likely to meet PA guidelines than those without disabilities (13, 14). These findings align with another study conducted in the United States, where half of the adults were physically inactive and more likely to have a chronic disease (15–17). According to Martin Ginis et al. (5), the significant disparities in participation rates and the near-absence of population-level data on PA in people with disabilities from low-income and middle-income countries represent a problem for healthcare systems and population quality of life. In this regard, more extraordinary global efforts towards international goals such as the United Nations (UN) Sustainable Development Goals (SDG) of healthy lives and well-being for all have been directed to face these challenges. In this sense, the Latin American region is an active member state responsible for covering and accomplishing the SDG (18). One study analyzed the participation in PA of people with chronic health physical conditions (e.g., chronic back pain, angina, arthritis, asthma, diabetes, hearing problems, tuberculosis, visual impairment, and edentulism) in a few South American countries (i.e., Brazil, Ecuador, Paraguay, and Uruguay) (19). Even though this study provides novel information about a multi-national scope, people with disabilities and different impairments (i.e., hearing, intellectual, or mixed-related disabilities) were not incorporated into the analysis.

The 2020 Chilean National Physical Activity and Sports Habits in Populations with Disabilities study (CNPASHPwD; Estudio Nacional de Hábitos de Actividad Física y Deporte en Población con Discapacidad in Spanish) (20), aims to collect information and epidemiological evidence through the application of a national survey about patterns of practice during coronavirus disease 19 (COVID-19) pandemic. However, to favor fewer infection rates during the pandemic, international organizations and governments imposed various preventive measures, such as restricted movement of the citizens, social/physical distancing, and banning social gatherings (21). It is essential to highlight that people with disabilities are especially exposed to the effects of the pandemic, where confinement strategies drastically decreased PA levels and increased sedentary lifestyles even more, which directly affected their health parameters (22, 23). Exploring the prevalence of PA levels considering participants with the mentioned health conditions and socio-demographic data from a country in the South American region may provide novel knowledge and bridge gaps in local factors that could facilitate intervention strategies targeting the population with a disability.

Therefore, this study aimed (i) to describe the prevalence of reported PA levels of Chilean adults with disabilities and (ii) to examine the associations of their PA levels with socio-demographic factors and type of disability participating in the 2020 CNPASHPwD during the COVID-19 pandemic.

## 2. Materials and methods

### 2.1. Study design and participants

The 2020 CNPASHPwD study used a cross-sectional design to identify PA levels and competitive sports practices in the national population with disabilities completed during the COVID-19 pandemic (20). A stratified cluster and multistage random sample from the Population and Housing Census of 2017 was selected, considering individuals aged ≥13 years with a disability, a valid home address from all socio-economic sectors, and geographical units in urban and rural sectors. Sample size was calculated for each region, according to estimating proportions formulae assuming *p* = 0.5, setting the statistical power at a 95% confidence interval and a sampling error of 5.9%. With this calculation, the sample size required in each region ranged between 269 and 276 participants (24). Data were collected by census track covering households using face-to-face questionnaires. The planned sample size was 4,393 surveys, but only a response rate of 87% was achieved, corresponding to 3,833 surveys (20). The surveys were completed in all the regions from November 21 to December 23, 2020. However, two southern regions were in partial restrictions, limiting the possibility of reaching the planned sample size. For participants whose disability prevented them from answering the surveys independently (e.g., intellectual disabilities), their parents/guardians were instructed to support their responses. For this study, only adult participants aged ≥18 were considered for the analysis (25), with a final sample of 3,150 participants. The study protocol was reviewed and approved by the ethics research committee of the Ministry of Sports and carried out following the guidelines of the World Medical Association’s Declaration of Helsinki. All participants gave consent to participate.

### 2.2. Variables

#### 2.2.1. Socio-demographic

The survey includes questions to collect information about age, gender (male, female, or no answer), socio-economic status (i.e., five levels according to income *per capita* in quintiles), area of residence (urban or rural), and region of residence denominated according to the number of the administrative divisions (north: XV, I, II, III and IV; central: XIII, V, VI, VII, VIII; and south: XVI, IX, X, XI, XII, XIV).

#### 2.2.2. Disability types

Respondents were categorized into five disability types according to their impairments, including physical, visual, hearing, intellectual, and mixed which generate activity limitations and restrict their daily participation according to the International Classification of Functioning, Disability, and Health framework, which is in line with the UN definition of disability (18, 26).

#### 2.2.3. Physical activity

Physical activity was measured with the categorical option from the validated Swedish National Board of Health and Welfare, which has an acceptable correlation with objective devices among adult the population (27, 28). Duration of moderate or vigorous-intensity leisure-time PA was reported according to time intervals for all seven days of the week. Intervals included 0 min/day, < 15 min/day, 15–30 min/day, 31–45 min/day, 46–60 min/day and > 60 min/day. The average minutes of each interval were truncated to create categories of recommendations according to the WHO (29), into 0 min/week (nonphysical activity), 1–149 min/week (some but less than recommended), and ≥ 150 min/week (active).

### 2.3. Statistical analysis

Descriptive characteristics variables are presented with mean and standard deviation for continuous variables, while frequency and percentages for categorical variables. Bivariate associations using *t-*test, Chi-squared, or Fisher exact test assessed for differences among PA levels. Multivariable logistic regression was performed to analyze the association between PA recommendations (active ≥150 min/week or inactive <150 min/week) and disability type, gender, age, socio-economic status, area, and region of residence. The results from the logistic regression were expressed in odds ratios (ORs) and their 95% confidence intervals (95% CIs) for crude (disability type) and adjusted models (all covariates). All analyses were undertaken using Stata Version 15.1 (Stata Corp, College Station, Texas, United States), and a complex sample design was used. A *p*-value of <0.05 was considered statistically significant.

## 3. Results

In total, 3,150 participants were considered for this analysis. The participants’ socio-demographic characteristics and PA levels are described in Table 1. Of the participants, 59.8% were female, 52.9% reported visual impairments or disability, 50.8% belonged to the two lowest socio-economic quintiles, 44.3% resided in the central region, and 82.9% lived in urban settings. Regarding PA levels, only 11.9% of the participants were physically active.

**Table 1:** Socio-demographic characteristics of participants.

Variable	Unit	Mean	SD^a^
Age	Years	47.2	18.9
	**Category**	***n***	**%**
Gender	Male	1,250	39.7
	Female	1885	59.8
	No answer	15	0.5
Disability type	Physical	680	21.6
	Visual	1,667	52.9
	Hearing	228	7.2
	Intellectual	117	3.7
	Mixed	458	14.5
Socio-economic status	ABC1 (wealthiest)	214	6.8
	C2	512	16.3
	C3	823	26.1
	D	1,028	32.6
	E (poorest)	573	18.2
Area of residence	North (XV, I–IV)	1,113	35.3
	Center (MR, V–VIII)	1,366	43.4
	South (IX–XII, XIV)	671	21.3
Zone	Urban	2,610	82.9
	Rural	540	17.1
Physical activity levels	None	1971	62.6
	<150 min/week	803	25.5
	≥150 min/week	376	11.9

The area of residence is denominated according to the number of the administrative division (I-XV) and MR, metropolitan region.

a Standard deviation.

Table 2 illustrates the prevalence of PA according to the socio-demographic characteristics of the participants with disabilities. Those who reported meeting PA recommendations were younger than their counterparts (*p* < 0.001). According to gender, larger proportions of males were classified as active compared to female participants (*p* < 0.001). There was a particular higher prevalence of non-practice of PA among those participants with physical (23.6%), intellectual (4.1%), and mixed (16.2%) disabilities (*p* < 0.001). Physical inactivity prevalence increased in participants from the poorest quintiles (i.e., E: 20.8%), while most active participants were categorized in the wealthiest group (i.e., A: 12.5%). A lower prevalence of participants from the northern region achieved ≥150 min/week (27.1%) in comparison to participants from the central area (47.3%). Nearly 80% of participants from urban areas fulfilled the PA recommendations.

**Table 2:** Prevalence of physical activity levels in participants according to their socio-demographic characteristics.

Physical activity levels		0 min/week *n* = 1,971 (62.6%)	<150 min/week *n* = 803 (25.5%)	≥150 min/week *n* = 376 (11.9%)	
Variable	Unit	Mean (SD^a^)	Mean (SD^a^)	Mean (SD^a^)	*p-*value
Age	Years	49.9 (18.9)	43.2 (18.3)	41.8 (17.6)	< 0.001
	**Category**	***n* (%)**	***n* (%)**	***n* (%)**	
Gender	Male	749 (38.0)	307 (38.2)	194 (51.6)	< 0.001
	Female	1,214 (61.6)	490 (61.0)	181 (48.1)	
	No answer	8 (0.4)	6 (0.8)	1 (0.3)	
Disability type	Physical	466 (23.6)	152 (18.9)	62 (16.5)	< 0.001
	Visual	990 (50.2)	450 (56.0)	227 (60.4)	
	Hearing	114 (5.8)	83 (10.3)	31 (8.2)	
	Intellectual	81 (4.1)	27 (3.4)	9 (2.4)	
	Mixed	320 (16.2)	91 (11.3)	47 (12.5)	
Socio-economic status	ABC1 (wealthiest)	101 (5.1)	66 (8.2)	47 (12.5)	< 0.001
	C2	270 (13.7)	170 (21.2)	72 (19.2)	
	C3	500 (25.4)	217 (27.0)	106 (28.2)	
	D	690 (35.0)	228 (28.4)	110 (29.3)	
	E (poorest)	410 (20.8)	122 (15.2)	41 (10.9)	
Area of residence	North (XV, I–IV)	787 (39.9)	224 (27.9)	102 (27.1)	< 0.001
	Central (MR, V–VIII)	782 (39.7)	406 (50.6)	178 (47.3)	
	South (IX–XII, XIV)	402 (20.4)	178 (47.3)	96 (25.5)	
Zone	Urban	1,614 (81.9)	666 (82.9)	330 (87.8)	0.021
	Rural	357 (18.1)	137 (17.1)	46 (12.2)	

The area of residence is denominated according to the number of the administrative division (I-XV) and MR, metropolitan region.

a Standard deviation.

**Figure 1:**
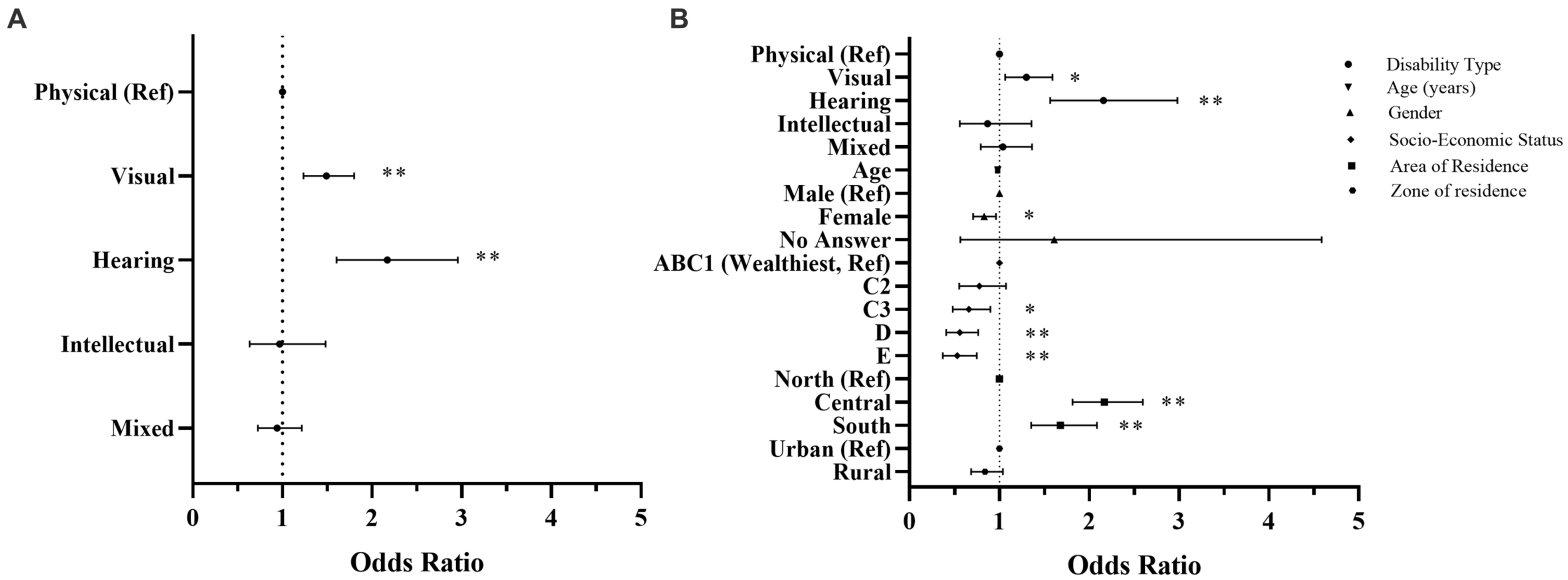
Forest plot of odds ratios and 95% confidence intervals. The plot represents the association between physical activity levels and the type of disability of the participants. **(A)** is adjusted for disability types and **(B)** is additionally adjusted for age, gender, socio-economic status, area of residence, and zone of residence. Results are interpreted as higher or lower odds of being physically active. Ref, reference; *p < 0.05; **p < 0.01.

The ORs and the 95% CI reported PA levels according to the socio-demographic characteristics of the participants can be found in Figure 1. Participants with visual and hearing impairment (OR = 1.30–2.16; 95% CI: 1.233; 2.957) were associated with higher odds of achieving international recommendations of PA than those with physical impairment. Also, older participants had fewer odds of being categorized as physically active (i.e., ≥ 150 min/week) compared to younger participants (OR = 0.98; 95% CI: 0.978; 0.987). In addition, female participants were less likely to be physically active than male participants (OR = 0.83; 95% CI: 0.709; 0.965). Moreover, participants who belong to the lowest quintiles were less likely to meet current PA recommendations (OR = 0.53–0.66; 95% CI: 0.371;0.901). Finally, participants living in the central or southern regions of the country were more likely to participate in adequate levels of PA than those living in the northern area (OR = 1.68–2.17; 95% CI: 1.355; 2.598).

## 4. Discussion

To the best of our knowledge, this is the first study to describe the prevalence of PA patterns and socio-demographic characteristics in a sample of Latin American adults with disabilities during the COVID-19 pandemic. The current study showed that 11.9% met the PA guidelines, and 62.6% declared non-physical activity. Moreover, males, younger, with visual or hearing disabilities from the wealthiest economic status and with residency in the central and southern zones of the country, were more likely to meet adequate levels of PA recommendations.

According to PA levels, these results align with previous studies, showing that people with disabilities reported lower levels than those without disabilities (14, 30). Furthermore, this can be seen in a recent work performed on South American adults during the COVID-19 pandemic lockdown showed that 56.9% of the participants were categorized as physically active, a higher prevalence compared to the reported in the present study (31). In addition, McGuire et al. (32) described that adults with disabilities were less likely to engage with recommended levels of PA in comparison to their counterparts, showing the health disparities which could be involved in this population and higher odds of developing chronic diseases (17, 33). Furthermore, previous reports showed that people with disabilities could also achieve health benefits by performing PA at levels even below international recommendations (5, 34), considering the particularities of the specific disability (35, 36). In this line, PA has been demonstrated as a feasible tool for improving and maintaining health in these groups, wherein every minute counts (29); however, compliance with PA recommendations is still low, corresponding to the reported by the participants. The update of the WHO guidelines on PA and sedentary behavior includes recommendations for adults living with a disability, facilitating the development and implementation of policies for participation in this population. However, considering the extensive range of health and functional profiles, a broader level of PA recommendation is needed according to each group’s characteristics as the evidence available increases (12). Additional challenges could be faced by people with disabilities during measures of social isolation, whereas previous studies reported that the use of assistive devices and environmental barriers limit PA participation (9, 11, 37), and the impact of the housing-built environment on the quality of life (38).

Some authors suggested that along with the natural aging process, adults with a disability could experience more difficulties associated with an accelerated musculoskeletal impairment-specific process, secondary health conditions, and lower levels of PA that affect overall health and quality of life (39–42). In this context, PA is critical to maintaining or improving physical functioning in people with chronic health conditions (43). According to age, the results indicated that lower PA levels were presented in major proportions among older adults. Previous studies have shown that older adults without disabilities are more likely to be inactive and present an increased cardiovascular risk than younger adults (44, 45). Moreover, higher amounts of sedentary behavior time could negatively impact the increased risk of premature mortality, especially in physically inactive individuals, reinforcing the importance of maintaining higher levels of total PA at any intensity (46, 47). The public health crisis increased the risk of death and severe outcomes due to COVID-19 in older people living with disabilities more than in people without disabilities, significantly impacting the disability community and probably affecting their levels of PA (48).

In terms of gender, female participants presented a higher prevalence of physical inactivity, in line with previous studies among women with disabilities (49–52). Women with disabilities are at higher risk of inactivity-related health consequences due to facing significant barriers in different spheres and difficulting participating in PA (5, 53). Especially in Latin America, data from people without disabilities described that women’s participation in PA had been identified as insufficient and lower than in other regions of the world. This concern reinforces the need to develop specific strategies for this population (53, 54).

Previous reports informed that people with multiple disabilities present the lowest PA levels probably due to the number of barriers at different levels (i.e., psychological, environmental factors, activity costs, and lack of support) and difficulties that are determinants of participation (49, 55). In addition, Lobenius-Palmér et al. (50) assessed the prevalence of PA in youths with multiple disabilities and reported that participants with hearing impairments were the most physically active. This finding is consistent with the results found in this study, where participants with hearing and visual impairments present higher levels of PA than those from the physical disability group. Probably, these results could be influenced due to the specific-local barriers that face people with physical and intellectual disabilities, limiting their possibilities to engage in different types of PA and achieve optimal health (15, 39, 40). Additionally, the disability itself, the requirement of support personnel, and the use of assistive-specific devices to facilitate the practice of PA could limit the chances of opportunities for participation (9). People with disabilities face complex challenges where government responses do not fully address the population’s basic needs in a health emergency scenario such as the COVID-19 pandemic, hindering even more, the possibility of being concerned about the practice of PA (23). The pandemic measures drastically impact the PA levels and the increment of sedentary behaviors in people living with disabilities (56) and able-bodied populations (57), a cautionary aspect that should be considered when interpreting the results.

People with disabilities experience higher living costs due to difficulties with functional autonomy, support requirements, and access to specific equipment, facing more significant inequalities than people without disabilities, summating in a considerable socio-economic disadvantage (58–60). It is remarkable to describe that the disability prevalence, on average, is higher in low-and lower-middle-income countries than in upper-middle-and high-income countries (59). Particularly, socio-economic status was identified as a relevant factor that influences the time engaging in PA and impacts the prevalence of sitting time, showing inequality patterns between the poorest and wealthy segments of the Chilean population (61). This study showed a higher prevalence of physical inactivity in the lowest quintiles. Similarly, previous research highlighted that the principal determinants in low socio-economic status communities to engage in PA participation involve the urban environment, financial constraints, work-life integration, community engagement, social support, and individual psychological factors (62). People living with disabilities are constantly constrained by barriers that difficult their involvement in PA and sports, where the economic factor could be relevant for accessing to sport, requiring specific equipment, or higher transport costs, among others (10, 11, 37, 63–65). Werneck et al. (65) described that socio-economic inequalities in South American populations without disabilities increased for total and leisure-time physical activity over the years. According to the author’s knowledge, there are no reports considering PA disparities according to the socio-economic characteristics of people living with disabilities in the Latin American region prior or during the COVID-19 pandemic.

The COVID-19 pandemic disproportionately affected the socio-economic lives, particularly the poorest and most vulnerable segments of society, such as women, the older adults, and people living with disabilities (48). The lack of adequate socio-economic support for the population with disabilities during the pandemic could have significant repercussions on daily life, considering the low levels of participation in the labor market and high levels of involvement in the informal economic employment in the region, constrained by health measures limiting all type of activities (66, 67). Our results demonstrated greater physical activity levels in people living in the central and southern regions of the country when compared to those from the northern regions. These results are probably conditioned by the country’s geographical diversity, where the Chilean population is exposed to high levels of urbanization, principally in the central zone (68). In particular, Sadarangani et al. (69) showed that in the Chilean non-impaired population, the same differences in PA level were presented according to the region of residence, reinforcing the particularities of each environment and the possible impact on health parameters. Moreover, local disability-specific policies and consistent actions promoting physical activity in people with disabilities are important to address greater participation levels advocating this aspect as a basic human right. Although Chile is a member of the UN, and there are some policies and actions oriented specifically to this population segment, the results of this study suggest relevant disparities due to the high prevalence of physical inactivity, remarking that these strategies are not enough (70). Furthermore, people with disabilities could experience more barriers and fewer opportunities to access services or infrastructure. Consequently, they participate less in PA in rural contexts, facing more challenges than their counterparts in urban residency zones (71). Lastly, cultural-specific characteristics could also constraint the participation of people with disabilities, considering that some local barriers may be relevant only in the study country and not in other regions, impacting in a unique form the complex relationship between barriers and facilitators for engagement in physical activity (72, 73).

### 4.1. Strengths and limitations

According to Martin Ginis (5), there is a need to develop research on PA in people with disabilities, focusing on data collection in middle and low-income countries where monitoring is required to measure the accomplishment of the UNs´2030 Agenda and SDGs. The novelty of this research relies on the analysis of a large random sample of participants living with disabilities, providing data on a population from the Latin American region, which could help to have a more profound understanding of the magnitude of the prevalence of PA levels in people with disabilities. Facilitating their participation in PA could be a challenge that requires multi-sectorial efforts considering the particularities of this population and investing in inclusive environments that benefit all the society (12). A more holistic approach is necessary for further studies addressing other aspects that could impact PA participation and patterns, such as sitting time, screen time exposure, active transport, gender differences, or nutritional behaviors.

This study is not free of limitations that should be addressed. First, the PA levels reported in this study are based on self-reported methods, which could include estimation problems due to potential self-reporting bias. Second, CNPASHPwD employed a cross-sectional design, precluding inferences about causality. Third, the number of participants in each disability group is limited (i.e., intellectual and hearing impairments), and the degree of disability is unavailable; thus, residual confounding remains as a possibility. Also, the health measures to contain the COVID-19 pandemic could influence the responses of the level of participation in PA; however, this novel information is of interest considering the absence of previous reports. Further studies should consider these limitations and explore possible barriers and facilitators impacting PA patterns. In addition, future studies could incorporate longitudinal analysis, neighborhood countries, transport modes or distance to sports facilities, considering the multiple barriers faced by people with disabilities.

In conclusion, 11.9% of the people living with disabilities in Chile during the COVID-19 pandemic were classified as physically active, in which especially males and those from the highest socio-economic segments, and those living in the central and southern regions of Chile were more likely to be active. These data could be helpful to potential strategies or local policies to promote PA in certain specific disability groups in collaboration with different sectors of Chilean society and in concordance with the SDGs.

## Data availability statement

The data analyzed in this study is subject to the following licenses/restrictions: Transparency law. Requests to access these datasets should be directed to https://www.portaltransparencia.cl/PortalPdT/.

## Ethics statement

The studies involving human participants were reviewed and approved by Sports Ministry. The patients/participants provided their written informed consent to participate in this study.

## Author contributions

KS and MH involved in conceptualization and roles or writing—original draft. KS involved in data curation, performed the formal analysis and has full access to all the data in the study and is responsible for the decision to submit for publication. KS, MH, RR-C, CC-M, RR, CA, GF, and NA-F were involved in writing—review and editing. All authors contributed to the article and approved the submitted version.

## Conflict of interest

The authors declare that the research was conducted in the absence of any commercial or financial relationships that could be construed as a potential conflict of interest.

## Publisher’s note

All claims expressed in this article are solely those of the authors and do not necessarily represent those of their affiliated organizations, or those of the publisher, the editors and the reviewers. Any product that may be evaluated in this article, or claim that may be made by its manufacturer, is not guaranteed or endorsed by the publisher.
